# The Calculus of Committee Composition

**DOI:** 10.1371/journal.pone.0012642

**Published:** 2010-09-17

**Authors:** Eric Libby, Leon Glass

**Affiliations:** 1 New Zealand Institute for Advanced Study, Massey University, Auckland, New Zealand; 2 Department of Physiology, McGill University, Montreal, Canada; University of East Piedmont, Italy

## Abstract

Modern institutions face the recurring dilemma of designing accurate evaluation procedures in settings as diverse as academic selection committees, social policies, elections, and figure skating competitions. In particular, it is essential to determine both the number of evaluators and the method for combining their judgments. Previous work has focused on the latter issue, uncovering paradoxes that underscore the inherent difficulties. Yet the number of judges is an important consideration that is intimately connected with the methodology and the success of the evaluation. We address the question of the number of judges through a cost analysis that incorporates the accuracy of the evaluation method, the cost per judge, and the cost of an error in decision. We associate the optimal number of judges with the lowest cost and determine the optimal number of judges in several different scenarios. Through analytical and numerical studies, we show how the optimal number depends on the evaluation rule, the accuracy of the judges, the (cost per judge)/(cost per error) ratio. Paradoxically, we find that for a panel of judges of equal accuracy, the optimal panel size may be greater for judges with higher accuracy than for judges with lower accuracy. The development of any evaluation procedure requires knowledge about the accuracy of evaluation methods, the costs of judges, and the costs of errors. By determining the optimal number of judges, we highlight important connections between these quantities and uncover a paradox that we show to be a general feature of evaluation procedures. Ultimately, our work provides policy-makers with a simple and novel method to optimize evaluation procedures.

## Introduction

Ever since the late 18th century, when Nicolas de Condorcet identified problems and paradoxes that arise when combining the opinions of independent judges [1], it has been clear that it is difficult, if not impossible, to establish evaluation procedures that result in fair and accurate decisions [2]–[4]. Yet, evaluation is at the heart of many societal procedures including: governmental decisions [5]–[8], peer-reviewed processes [9]–[19], and athletic competitions [20]–[22]. The recent revisions of evaluation procedures in areas as diverse as figure skating [21], [22] and NIH grant review [9]–[13] underscore both the inherent difficulties and the perceived importance of developing optimal methods.

In this article we do not examine the history of these issues nor discuss the extensive social science research concerning psychological and group dynamic aspects of decision processes [23]–[25]. Nor do we consider the well-studied problem of how to combine the evaluations of a panel of judges [2], [4]–[6], [8], [26]–[31]. Rather, we consider an important issue in the design of any evaluation procedure: how many judges should be used?

The Condorcet Jury Theorem states that if judges are equally accurate, perform better than random selection, and make decisions according to majority rule then increasing the number of judges will always result in more accurate evaluations [1]. Accuracy, however, cannot be the only criterion for designing an evaluation procedure. For example, if a scientific journal needs to determine whether a paper should be published then consulting a large number of reviewers is not practical, even if it could lead to more accurate decisions. So despite the importance of accuracy, there is also an issue of cost whether in time or money or both.

We distinguish between two types of costs: the cost of a wrong decision and the cost of a judge. If all options were equally valuable then there would be no reason to consult any judges. The use of an evaluation procedure, therefore, must assume that there is at least one option better or “correct” among the choices. In this context, picking an inferior option incurs some type of cost whether it is lost revenue or greater risk to financial loss. Although it may be difficult to determine the “correct” choice(s) or the precise costs, societal institutions make major efforts to set evaluation procedures. Difficulty in determining whether the outcome was the best possible should not preclude examination of the relevant factors at the heart of all evaluation procedures. While it is beneficial to avoid a costly mistake, judges also have associated costs in the form of expenses or salaries. The optimal number of judges must balance these costs, weighing the benefit of additional judges against their cost.

Previous studies have mainly addressed the question of how best to select among competing options using a set number of judges [2], [4]–[6], [8], [26]–[31]. Though there has also been some consideration of the accuracy and cost of an evaluation procedure as a function of the number of judges [9], [15], [32]–[34], these earlier papers did not explicitly consider the cost of making a wrong decision in their calculations of the optimal number of judges. The cost of an error is important because it converts the accuracy of an evaluation into a currency comparable to the cost of judges. Here, we examine how the optimal number of judges depends on the accuracy of the judges, the cost of the judges, the cost of errors, and the method for combining the judges' scores.

Throughout the paper, we assume that judges are honest but prone to error. In this case, we can define a judge's accuracy as the expected probability that an individual judge will choose the correct option (see File S1 for a glossary of additional key terms). This definition of accuracy may be case-specific and subject to other qualifications but there has been work in estimating judge accuracy in complex scenarios such as grant review [14]–[16], [19]. Some evaluation procedures such as Cooke's method attempt to estimate judge accuracy with seed questions as part of the procedure [26]–[28].

If we have some measure of judge accuracy and know the rules for combining the evaluations of the judges, we can compute a curve that gives the probability of making a correct decision as a function of the number of judges: *the judge accuracy curve*. The judge accuracy curve can be computed using many different models of evaluations including ones in which judges are unequal, non-human, or reach decisions under influence from other judges. With the judge accuracy curve, the cost per judge, and the cost per error, we can compute the *cost curve*, which represents the expected cost as a function of the number of judges. The number of judges at the minimum of the cost curve is defined as the optimal number of judges. For a given method of evaluation, at the optimal number of judges there is an implied relationship between the accuracy of the evaluation, the number of judges, and the ratio between the cost of an error and the cost per judge. Knowing any two of these quantities, allows us to estimate the third.

Based on our formulation we present a paradox. From the work of Condorcet, it might appear that if the accuracy of judges were improved, then the optimal number of judges should decrease. However, we show examples in which as the accuracy of the judges increases, the optimal number of judges may also increase. In the remainder of this paper we illustrate each of these points and present some practical implications of these results.

## Results

To balance cost and accuracy, we represent the expected total cost of an evaluation (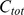) as
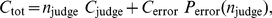
(1)where, 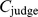 is the cost per judge, 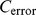 is the cost of making an incorrect decision, 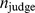 is the number of judges, and 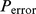 is the probability of making an error. 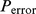 is a function of the number of judges and is equal to (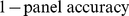), where panel accuracy is the probability a set of judges picks the better option. In practice 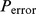 depends on many other factors but we hold these constant here. By taking a difference approximation of the derivative in Eq. (1), the optimal number of judges will occur when
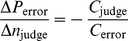
(2)where 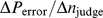 is the rate of change in 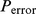 as a function of the change in the number of judges. In order to determine the optimal number of judges for particular situations, we first consider how 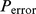 depends of the number of judges for the two most common evaluation methods: majority rule and scoring systems.

### Majority Rule

**Figure 1:**
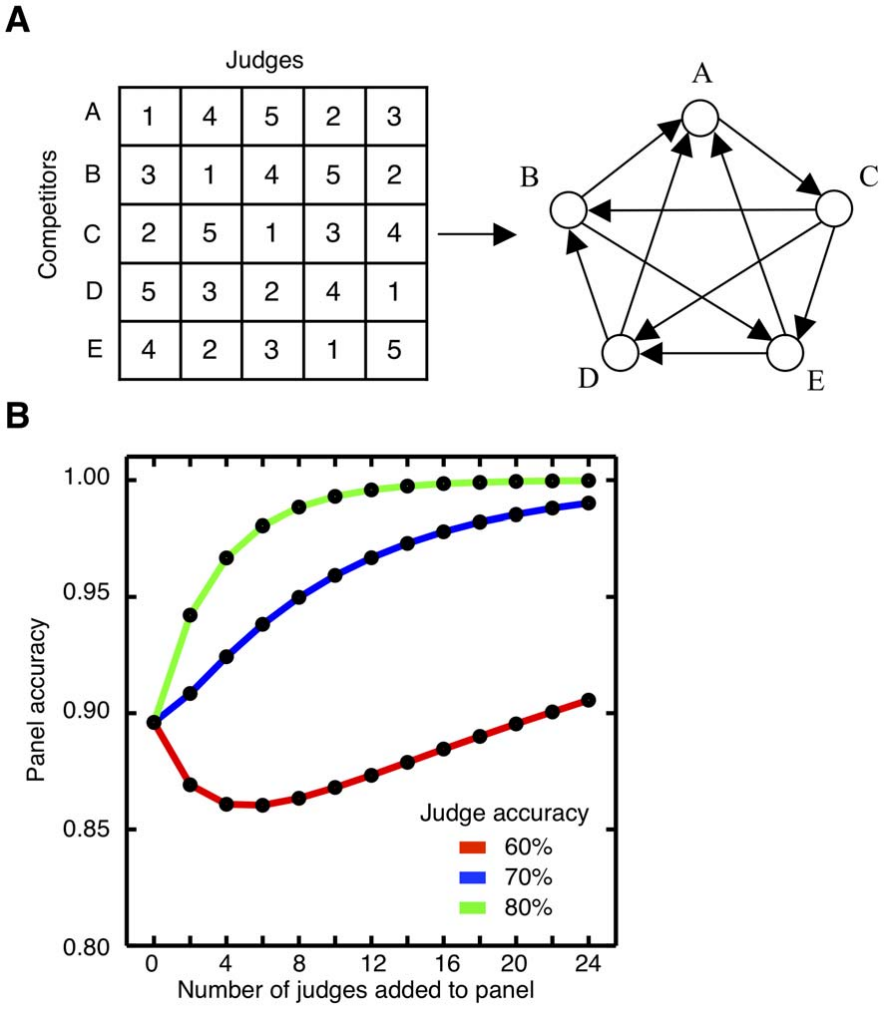
Condorcet results. (A) Voter's paradox. A hypothetical ranking of 5 competitors evaluated by 5 judges, where 1 is most preferred and 5 is least. By comparing options two at a time with majority rule, we produce a directed graph. The nodes are the competitors and the edges stemming from a node indicate which competitors that node beat. For example, the edge from B to A represents more judges favored B to A. The cycle through all of the nodes is indicative of the Voter's paradox (3): no clear winner. (B) The Condorcet Jury Theorem. Given a panel of 3 judges of 80% accuracy, we show the effects of adding judges with accuracy of 60% (red), 70% (blue), 80% (green) on the group's accuracy (panel accuracy) under majority rule. According to the Condorcet Jury Theorem if all judges have the same accuracy (green), then adding more judges increases the panel's accuracy. However, if the judges have a lower accuracy, the judge accuracy curve is not necessarily monotonic. The 60% accurate judges initially detract from the panel's accuracy and do not improve it until 22 judges are added (25 total in the panel).

In *majority rule* the option favored by the most judges is the winner. When the majority rule is used in a series of decisions like rank-ordering options, the well-known Voter's paradox can occur, so that there is no clear winner (Figure 1A) [3]–[6], [8], [20], [23]–[25]. Since majority rule gives an equal weighting to all judges, it does not make allowances for differences in judge confidence or accuracy [35], [36] – giving more weight to more accurate judges would improve the accuracy of the evaluation [27]. The Condorcet Jury Theorem shows that for equally accurate judges the judge accuracy curve monotonically increases as the number of judges increase. However, in practice the most accurate judges are consulted first.

To assess the effects of adding inferior judges to the judge accuracy curve, we suppose there is a panel of three judges deciding between two options A and B, where A is better than B. If there are 3 judges, each with 80% accuracy who make decisions independently by a majority rule, then their collective accuracy or the “panel's accuracy” is 89.6%. If we now add additional evaluators with 70% accuracy then the probability of making a correct decision increases even though the judges are inferior. If instead we add evaluators with 60% accuracy, the probability of a correct judgment decreases and does not increase until we add 22 judges, see Figure 1B (see Methods: Judge Accuracy Curve with Inferior Judges). These observations are consistent with earlier studies that showed that additional judges can increase or decrease a panel's accuracy depending on the accuracy of the individual judges and the number already in the panel [32]–[34]. Thus, judge accuracy curves need not be monotonic. This permits the possibility for multiple values of the optimal number of judges where the same expected total cost could be achieved with different numbers of judges and different accuracies of the evaluation.

### Scoring Systems

**Figure 2:**
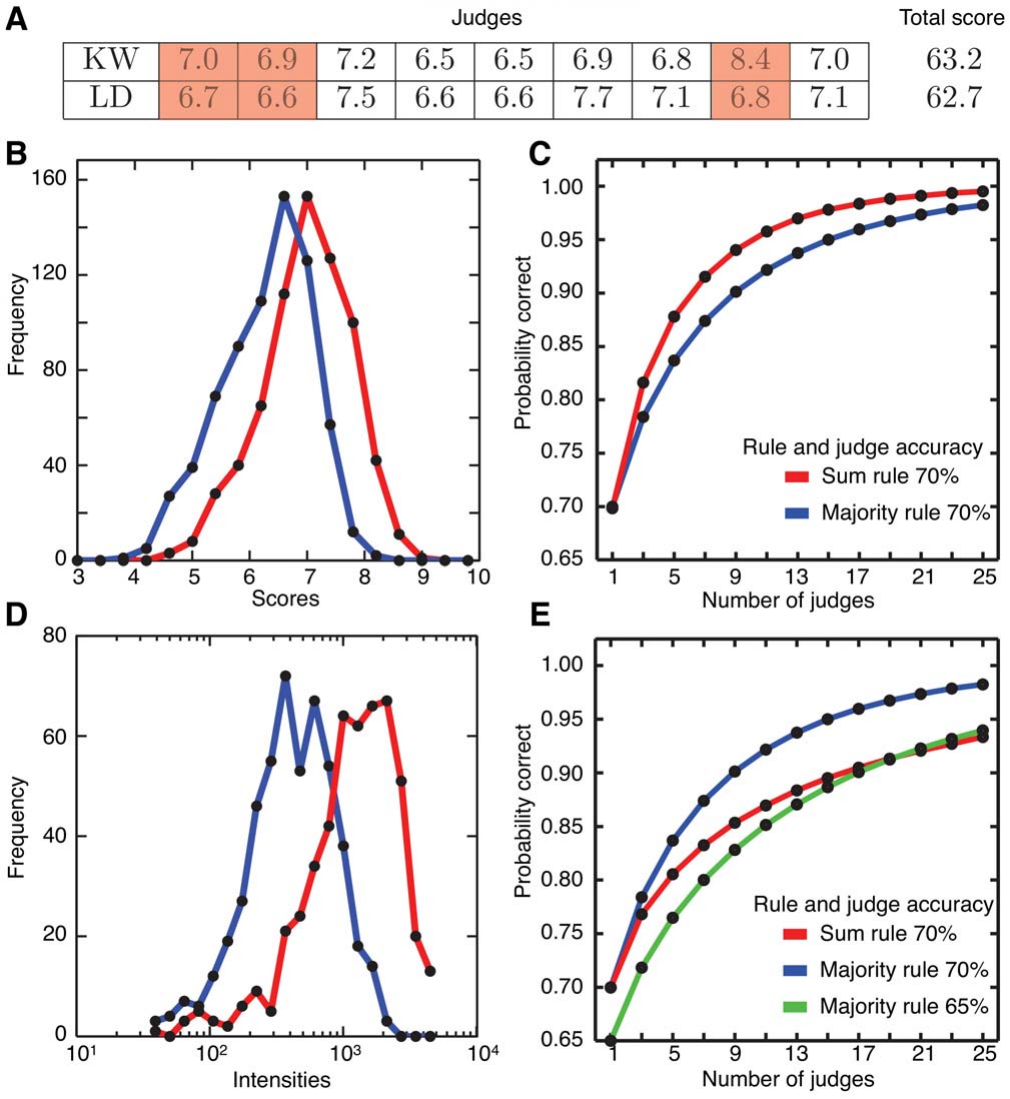
Majority rule versus sum rule. (A) The score-sheets for two competitors in the 2006 US Junior Figure Skating Championship show that while only 3 of the 9 judges (shaded) preferred Competitor KW, she has the higher total score. (B) The histograms show the frequency of scores for skaters ranked 1–5 (red) and those ranked 6–10 (blue). (C) Judge accuracy curves of the sum rule (red) and the majority rule (blue) on a hypothetical problem based on B with judges that are 70% accurate (see Methods). The sum rule is more accurate than the majority rule (confirmed by a nonparametric sign test, p-value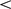.01 for all cases of more than one judge). (D) The distributions of probe intensities from the Affymetrix Latin Squares data-set measuring 64 picomolar (blue) and 128 picomolar (red) transcripts are very broad. (E) Judge accuracy curves on a model based on D (see Methods) where the judges' scores come from a wide scoring distribution. The majority rule of judges 70% accurate (blue) outperforms the sum rule of 70% accurate judges (red). In fact a majority rule of judges at 65% accuracy (green) does better than the sum rule of 70% accurate judges as the number of judges increases beyond 21. A nonparametric sign test confirms the differences in performance (p-value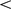.01) for all cases of more than one judge.

In contrast to the majority rule, *scoring systems*, such as those used in figure skating [22] are procedures in which judges assign numerical ratings, or scores, to competing options. The winner is often chosen by the sum rule whereby the option with the highest sum of scores wins. The sum rule can reach different conclusions than the majority rule (Figure 2A). Based on the distribution of scores in the US Junior Figure Skating Championship 2006 (Figure 2B, data available at http://www.usfigureskating.org), we simulate an evaluation and find that the sum rule is more accurate than the majority rule (Figure 2C and Methods: Sum Rule vs Majority Rule Methods). This finding supports the International Skating Union's switch from a majority rule-based system in 2002 to a sum rule-based system in 2006 [21], [22]. The sum rule enables judges' scores to reflect their certainty – so that a judge who scores two options with a 6.0 and a 9.0, respectively expresses more confidence than a judge who scores the same two options with a 6.0 and a 6.1, respectively. Adding the judges' scores gives the judges who assign larger score differences between options more power in determining the final evaluation. This can make scoring systems susceptible to manipulation by dishonest judges [2].

Although the example of Figure 2C shows a case in which a scoring system is more accurate than majority rule, the situation can reverse in instances where the judges' scores are widely distributed. An example of this is in gene microarray analysis [37], [38]. Genetic microarrays measure the expression of thousands of genes simultaneously using DNA probes. Each gene's mRNA transcript has a set of probes designed to bind it specifically in different regions. Samples of mRNA are labelled so that the amount bound to each probe on the microarray can be measured. Here, the probes are judges and the fluorescent intensities are scores. Applying the sum rule in this case is analogous to comparing means, except the sum rule does not consider the standard deviation nor calculate a test statistic like a p-value. In microarray analysis, the fluorescence magnitudes from the probes are broadly distributed (Figure 2D, data available at http://www.affymetrix.com/support/technical/sample_data/datasets.affx) unlike the tight distributions found in figure skating. Now the sum rule is not as accurate as a majority rule (Figure 2E) because each judge/probe is scoring according to a different rubric, i.e. the same score difference does not represent the same degree of confidence.

The distribution of the judges' scores affects the accuracy of the evaluation. In evaluations using scoring systems, guidelines are often used to ensure that the range of each evaluators' scores will be comparable. When that is not possible, as for microarrays, where the distribution of fluorescent intensities of individual probes is approximately lognormally distributed, it is common to first take the logarithm of the probe intensity before further statistical analysis [38], [39]. Hence, even with the same decision method (the sum rule) and the same judge accuracies the implementation of evaluation procedures such as the permitted scores can alter the judge accuracy curve.

### Paradox

**Figure 3:**
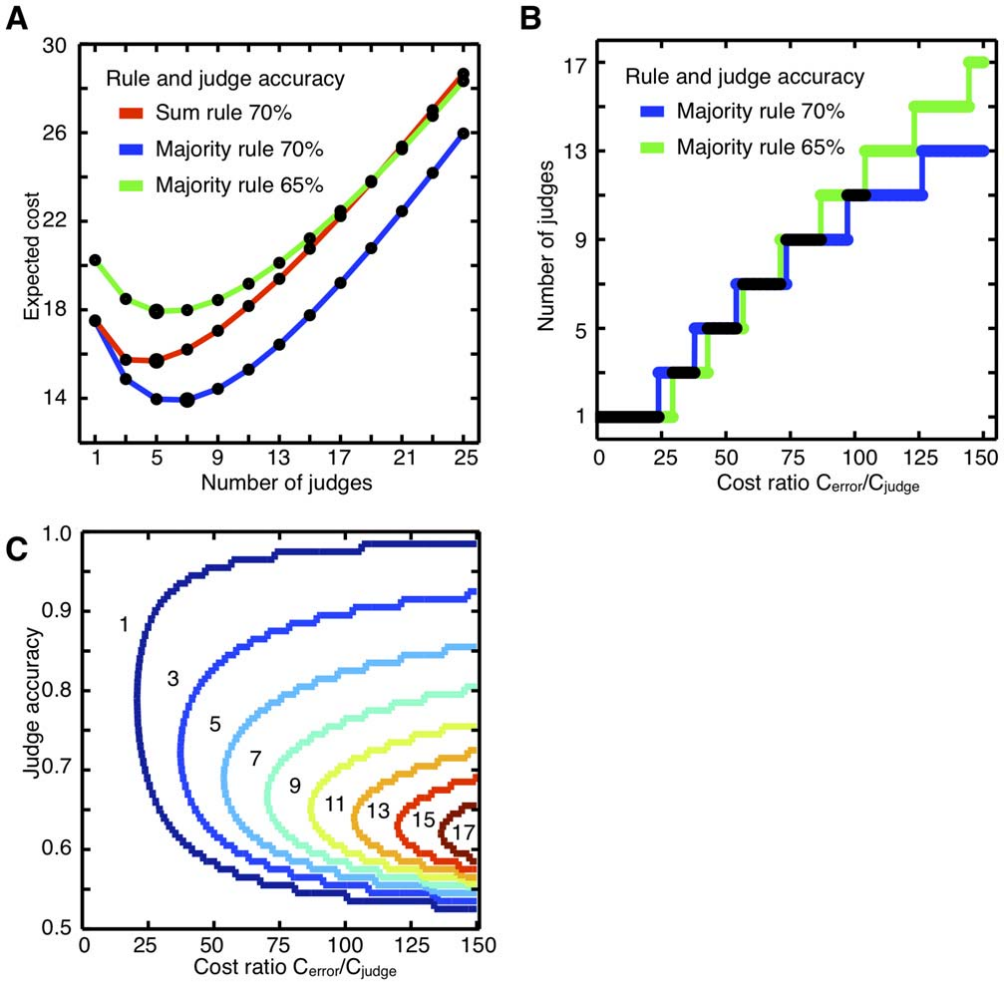
The paradox of the optimal number of judges. (A) The expected cost function (Equation 1) applied to the judge accuracy curve shown in Figure 2E. The cost per error is 55 times the cost per judge. The black line is the sum rule with 70% accurate judges, while the gray lines are majority rule (solid for 70% accurate judges and dashed for 65% accurate judges). The optimal number of judges, shown with the bigger dot, is different for the majority rule with 70% accurate judges. (B) The optimal number of judges as a function of the cost ratio. The majority rule with 70% accurate judges (red) and 65% accurate judges (blue) both increase the optimal number of judges as the cost ratio increases but diverge at points. (C) Contour plot of the optimal number of judges for majority rule as a function of the cost ratio and the judge accuracy. The reds indicate higher number of judges while the blues indicate lower numbers.

Based on the preceding analysis of the judge accuracy curve, we return to the cost analysis. Equation 2 shows that if we know the ratio between the cost per judge and the cost per error we can calculate the optimal number of judges for particular evaluations. For example, if we assume the cost per error is 55 times the cost per judge and use the 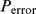 from Figure 2E we obtain Figure 3A. The expected total cost has a minimum at either 5 or 7 judges depending on the evaluation method and the accuracy of the judges.

Figure 3A presents a paradox: the optimal number of judges may increase despite the use of more accurate judges or more accurate evaluation methods. Even though the majority rule with judges 70% accurate is more accurate than both a sum rule with 70% accurate judges and a majority rule with 65% accurate judges, it has a minimum cost at 7 judges while the latter procedures have a minimum cost at 5 judges. This counterintuitive effect occurs because the judge accuracy curve for the majority rule with 70% accurate judges (see Figure 2E) has a larger slope than the other two procedures in the range of 5 to 9 judges. Thus, adding two additional judges reduces the probability of an error, and hence the expected cost, enough to offset the cost of the extra judges.

Since the optimal number of judges depends on the cost ratio 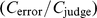, the paradox also depends on the relationship between the costs. Figure 3B shows that as the cost ratio increases the optimal number of judges increases divergently for majority rule procedures with 65% and 70% accurate judges. There are three ranges of the cost ratio where the majority rule with more accurate judges has a higher optimal number of judges. Once the cost ratio increases above 60, the more accurate procedure never has a higher optimal judge number.

The contours in Figure 3C denote the optimal number of judges of a majority rule evaluation as a function of judge accuracy and the cost ratio 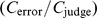. For small cost ratios, 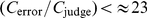, there is never an optimal panel size greater than one, because the cost of the judges outweighs the benefit of avoiding an incorrect decision. As the cost ratio increases so does the value of additional judges. For any optimal panel size of more than one judge, at the same cost ratio there are smaller optimal panels of both less accurate and more accurate judges.

Although Figure 3C was computed for a very specific evaluation procedure, we expect similar contour plots to occur over a broad range of evaluation procedures (see also File S1 and Figure S1). If the accuracy of each judge is no better than random selection or if the accuracy is 100% then the most cost effective strategy is to use only one judge. These two conditions define the boundaries on the contour plot and guarantee the generality of the paradox, even for a host of different evaluation settings.

### Cost Analysis

From the findings presented in Figure 3, there is an implied relationship between the cost ratio, the number of judges, and the accuracy of the evaluation. If we know any two of them, we can calculate a range for the third. To illustrate this, we consider different paradigmatic situations from athletic competitions and from academic grant review.

#### Athletic competitions

First, we apply our analysis to figure skating and infer the cost ratio from the number of judges and the expected accuracy of the evaluation. We estimate judge accuracy by assuming each judge is equally accurate and then counting the times a judge failed to give the stage's top competitor the highest score. While individual judges may have different accuracies, figure skating competitions occur in many different places with different collections of judges so such averaging approaches might be more appropriate to establish standards for evaluation procedures. From the results of the 2006 US Junior Figure Skating Championship, we calculate an individual judge's accuracy as 76%. This serves as an upper limit to the accuracy since it assumes the number one competitor was in fact the best. If the competition choice of nine judges is optimal, the implied ratio of cost of an error to cost of a judge is 100–152.

Now we use a similar approach to estimate the cost per judge in boxing bouts and compare with actual salaries. World Boxing Association bouts are scored by three judges. If the fight ends by knockout or is stopped by the referee then the scores are not used. If, however, the fight continues to the last bell then the judges' scores determine the winner. The scores are not added but instead count as a vote, so if two judges score one competitor higher that competitor wins regardless of the values of the scores. From this, we estimate a judge's accuracy as was done for the figure skating example. We counted how often a judge disagreed with the majority decision in 2006 (found at http://wbaonline.com) and calculated an accuracy of 95%. We set the cost of an error equal to the prize since the wrong person gets it. In boxing the actual cost of an error might reflect the displeasure of the fans and lost revenue as well as any legal ramifications. The prize varies depending on many factors, but for this analysis we set the prize to $100,000. Assuming three judges is optimal, we use the cost equation to calculate the range for a judge's salary as $ 305–$ 2138. This range is in line with the actual salary boxing judges get paid in a bout with a $100,000 purse (found at http://wbanews.com/artman/publish/regulations). The International Boxing Organization changes the fees paid to judges based on the purse (from http://www.iboboxing.com/public_disclosures.html). For prizes worth $100,000 judges get paid $1,200 which is in agreement with our calculated range. For prizes worth a million dollars, judges get paid $2,000, but using our analysis we calculate that the optimal number of judges would be 5 and not 3, suggesting that the procedure is not optimal.

#### Grant Review

Assuming a hypothetical situation that corresponds roughly to current norms of grant reviews, we estimate a reviewer's accuracy based on the costs and number of reviewers. To estimate the cost per judge, we calculate the cost per judge per review. We assume that a committee reviews 50 grants in two days. Assuming $1,000 in travel expenses per judge, we obtain a cost of 20 dollars per grant/per judge.

It is difficult to estimate the cost of an error in awarding research grants [16], [18], [19]. While publications may be a quantifiable measure there are issues concerning the quality of the publication, the role of coauthors, and the long range impact of the work [16], [19]. Although the cost of an error in grant reviews may be troublesome to exactly quantify, policy-makers are interested in the value of research [40]. In view of the uncertainty, we consider a range of costs per error from 1% to 100% of a $400,000 grant.

We consider the choice of ten judges to be optimal and from the cost equation find that: 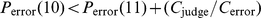 and 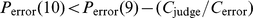. To find a reviewer's accuracy, we need a model of the decision-making process to see how 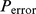 changes with judge accuracy.

Grant reviews typically employ a sum rule rather than a majority rule and use strict guidelines to set the range of judges' scores [9]. Because of this we used a model like the one from Figure 2C. Typically, grant reviews are a multi-stage process with alternating rounds of scoring and discussion [9]. Here, we apply our analysis to the first round of scoring before the reviewers discuss (“prescores”) and treat the reviewers as independent [9]. In File S1, we also consider models of dependency and multiple-stage processes.

For our 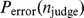 function we construct a scenario with two competing grants: A and B, where A is better than B. Each judge scores the grants according to a unique normal distribution 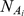 and 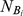 (where 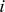 represents judge 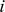
's score). The means of 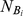 are samples from a normal distribution (mean of 2 with a standard deviation of 0.5) so each judge's average score is different. As in Figure 2C, the standard deviation of a judge's score for both A and B is a constant multiple of his/her mean score– 0.2 in this case. This ensures that the judges have the same coefficient of variation in their scores. The remaining term is the mean of 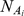 which determines the overlap of 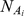 and 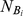, and consequently the extent to which a judge mistakenly scores B higher than A. For simplicity, we assume that the mean for 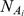 is a constant “c” times the mean of 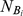, where 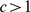.

To determine the implied accuracy of the judges before the discussion, we find the range of “c” values that give a 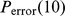 which meets the two criteria listed above. If the cost per error is 100% of the grant's value, or $400,000, the implied judge accuracy is 87–89%. If, on the other hand, the cost per error is only 1% of the grant's value, or $4,000, the implied judge accuracy is 67–69%.

## Discussion

Evaluation procedures are ubiquitous and great significance for individuals and society may devolve from a single decision. An important question in the design of any evaluation procedure is how many judges to consult. Previous work has approached this problem through analyzing evaluation costs or accuracy [14], [15], [32]–[34]. Here, we link these two components to find the optimal number of judges, balancing the cost of a judge with the cost of making an error in evaluation. Through this analysis, we demonstrate that there exists an inter-connectivity of the factors governing an evaluation procedure. Decisions such as whether to use a majority rule or sum rule or what type of scores judges can assign also affect the optimal number of judges. Thus, as evaluation procedures evolve over time so does the optimal number of judges.

We also found that paradoxically the optimal number of judges may be higher in evaluations using more accurate judges. From the cost analysis, better training of judges does *not* necessarily lead to a decrease in the optimal number of judges. Since the cost analysis is optimizing cost rather than accuracy, our result does not contradict the Condorcet Jury Theorem that states that for a fixed number of judges of equal accuracy, better training will always lead to improved group accuracy.

Our main results are summarized in Figure 3C, which shows the optimal number of judges, defined as the number of judges resulting in minimal expected cost, as a function of judge accuracy and 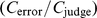 using a majority rule evaluation scheme. For judge accuracies near 50% or 100%, there is little benefit of adding additional judges, so that the optimal number of judges is 1. For a fixed 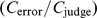, the optimal number of judges has a single peak for an intermediate value of judge accuracies. For example, for a 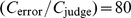 the maximal value for the optimal number of judges is 9 for judge accuracy in the range 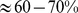. Although increasing the judge accuracy leads to a reduction in the optimal number of judges, paradoxically, decreasing the judge accuracy also leads to a reduction in the optimal number of judges.

Our theory can also be used to make quantitative predictions. Consider a granting institution that has a large variety of programs with different funding levels. If we assume that optimal evaluation procedures are being used, and that both the cost per judge and judge accuracy remain constant, computations such as those in Figure 2B predict the number of judges as a function of the size of the grant.

While the computations presented in this paper used simple models of evaluation procedures, our results should extend into more complicated evaluation procedures. In File S1, we apply our analysis to an example with judges of different accuracies who make decisions dependently. Ultimately, the cost analysis relies on the calculation of the probability of an error as a function of the number of judges (the judge accuracy curve). As long as we have a model of the decision-making process we can compute this curve for a variety of evaluation methods and conditions.

There are limitations to our approach that would require a more detailed and case-specific analysis. For example, if judges have different costs associated then a more elaborate cost analysis than the one presented here would need to be performed, assessing the costs and probabilities of an error with different combinations of judges. Our work also assumes that there is a model of the evaluation procedure and its parameters can be estimated. Thus, in grant reviews we assume that we know the procedures and can obtain some measurements of evaluation accuracy. This may be difficult in some situations– especially dependent decision-making where some reviewers may have more sway over committee members than others. Still, there is a body of work that attempts to estimate accuracy in areas such as grant review and it is possible for granting organizations to collect data necessary to build more explicit models [9], [14]–[16], [19].

Our work also relies on the assumption that it is possible to estimate the cost per error and the cost per judge. In cases like athletic competitions there is often a fixed fee for judges making the cost of a judge apparent. In contrast, for grant reviews judges are not usually remunerated monetarily and part of the cost of judges might include the time lost to other pursuits [17], [18]. Of course, there are often benefits for judging that accrue such as establishing ties with colleagues, editors, and grant administrators. The cost per error can also be difficult to explicitly quantify. For example, although, nominally the cost per error for a research grant review is a product of the probability of failure and the value of the grant, success in obtaining a grant may have major personal ramifications in terms of career advancement and monetary benefits such as patents or additional grants that might accrue or not depending on successful completion of a project. *However, a major point of the current analysis is that there is an implied range for* 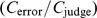 *that is set by the number of judges,* *Figure 3C*. This point should be appreciated by those who set evaluation procedures.

The findings presented in this paper are particularly relevant in the context of a recent review of NIH grant procedures [10] which highlighted the need for more explicit criteria for grant evaluation, reviewer training, and evaluation of reviewers. Although the NIH report also focused on the need to adopt procedures that would lessen the administrative burden, it also concluded (p. 78) “Engaging more reviewers per application and throughout the review process will help to ensure review quality and consistency, as would enhanced reviewer training.” However engaging more reviewers may not be advantageous if the reviewer quality of additional reviewers is inferior to a smaller, more expert panel, Figure 1B [32]–[34]. Even if increasing the number of reviewers increased the accuracy of the decision, it certainly increases the administrative burden which is a type of cost.

The focus of this paper has been finding the balance between accuracy and cost. By making explicit the relationships between the evaluation accuracy, the cost per judge, and the cost per error, the methods reported here should help policy-makers improve decision-making procedures.

## Methods

Computer code for Figures 1B, 2C, 2E, 3A, 3B, and 3C can be found in File S1.

### Judge Accuracy Curve with Inferior Judges

We can calculate the accuracy (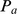) of a panel that uses the majority rule using equation 3 below. We assume that all judges are equally accurate and have a probability 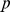 of choosing the right option. The variable 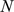 is the number of judges in the panel (we assume 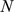 is odd to avoid ties). The panel's accuracy can also be expressed as the ratio of an incomplete beta function and a beta function.
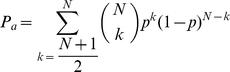
(3)


When we add additional judges with a different accuracy, we calculate the panel's accuracy with equation 4 [32]. This represents a mixing of two panels: 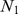 judges with accuracy 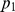 and 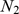 judges with 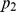 accuracy such that the total number of judges is odd.
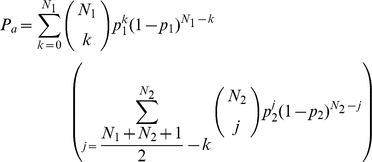
(4)


### Sum Rule vs. Majority Rule Methods

We first assume that there are two competitors A and B, where A is better than B. These competitors are scored by a number of different judges. An individual judge is not perfectly consistent and will some times give the same performance different scores. To account for this lack of precision, an individual judge's scores for A and B will be drawn from two different normal distributions 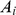 and 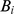 (where 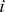 is an index for judge 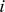). The mean of 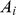 will be larger than the mean of 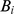 since A is better than B.

The amount of overlap between the 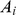 and 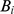 determines the inaccuracy of the evaluation, quantifying how often B is incorrectly given a higher score than A (judge accuracy = 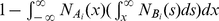). This is a common interpretation of scores in which if a judge gives the incorrect option a higher score, then the judge is wrong. For simplicity, each judge has the same accuracy, say 70%.

In addition to the distribution of a given judge's scores for a single competitor, different judges may assign scores for the same competitor based around a different mean value. So any two judges will have scoring distributions for A with different means. To simulate the differences between judges, the mean score each judge assigns option B is randomly drawn from a tight or broad distribution, modeled by a normal or a log normal distribution, respectively. Thus, while an individual judge scores options A and B according to normal distributions 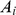 and 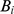, the mean of 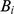 is picked from either a normal or lognormal distribution. We then determine the mean of that judge's 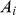 scoring distribution by multiplying the mean of 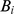 by a fixed constant 1.16 (the same for all judges throughout the simulation, calculated so the judges are 70% accurate). For simplicity, we also fix the the coefficient of variation for 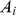 to be the same as 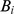 and equal to 0.2.

The total accuracy of a panel of judges in these simulations depends on the number of judges and whether their mean scores come from a tight or broad distribution. Similar to figure skating, the tight distribution of judges' scores for the mean of 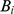 has a mean 7 and standard deviation .7 (used in Figure 2C). The log normal distribution (used in Figure 2E) has mean 700 and standard deviation 1400. Each data point represents the mean accuracy from 100,000 different samples of judges' scores (100,000 panels of judges).

## Supporting Information

File S1 This supplementary material, referred to in the text as File S1, includes a glossary, a worked example with added complexity, and computer code for the figures.
(0.15 MB PDF)

Figure S1 The vertical axis is the constant added to “c” for every judge, thus further separating the scoring distributions for the grants and improving each judge's accuracy. The horizontal axis is the cost ratio, (cost per error)/ (cost per judge). The colored bar shows the optimal number of judges. As in Figure 3C from the paper, there is a single peaked surface, and thus the paradox is present.
(0.15 MB TIF)
