# Supplementary material for: The Calculus of Committee Composition
Source: PLoS One. 2010 Sep 17;5(9):e12642. doi: 10.1371/journal.pone.0012642 (PMC2943248; doi:10.1371/journal.pone.0012642)
Supplement: File S1 — This supplementary material, referred to in the text as File S1, includes a glossary, a worked example with added complexity, and computer code for the figures. (0.15 MB PDF) [file pone.0012642.s001.pdf]

Supplementary material

Glossary

Key terms

- *correct decision* : the best of a set of competing options.
- *cost curve* : a graph of the total expected cost of a decision (on the vertical axis) versus the number of judges on the horizontal axis
- *cost of a judge* : the expected financial burden of a judge in an evaluation procedure.
- *cost of an error* : the expected financial burden of making the wrong decision in an evaluation procedure.
- *judge accuracy* : the expected probability that an individual judge will choose the correct option.
- *judge accuracy curve* : a graph that displays the panel accuracy on the vertical axis versus the number of judges on the horizontal axis.
- *majority rule* : a method for reaching a decision where the option supported by the most judges wins.
- *optimal number of judges* : the number of judges with the lowest total expected cost, the number of judges where the cost curve is at a minimum
- *panel accuracy* : the percentage of correct decisions made by a group of judges or the expected probability that a group of judges will make a correct evaluation based on past performance. Panel accuracy depends on many factors including the accuracies of the individual judges and the evaluation procedure.
- *panel error, probability of an error* : the probability that an incorrect decision is made. Probability of an error is equal to $(1 - \text{panel accuracy})$ when panel accuracy is expressed as a probability.
- *scoring systems* : evaluation procedures where judges assign numerical ratings to competing options to make a decision
- *sum rule* : a method for reaching a decision under a scoring system regime where the option with the highest aggregate (sum) of scores wins.

Grant Review Examples with Dependencies

In the grant review example presented under the *Cost Analysis* section, we only considered the pre-scores which were produced by reviewers independently scoring each grant. Here, we imagine that there is an additional stage of the grant review process such that the judges discuss their opinions and score again. This second scoring introduces an element of dependency. We use the same procedure as presented in the paper to generate the pre-scores and then investigate the effects of three different models of "consultation" on our inference of judge accuracy. We note that with two rounds of scoring there could be two measure of accuracy: before and after consultation. This, however, does not present a significant problem because with a model of the decision-making process we can convert between pre-discussion and post-discussion scores and calculate judge accuracy for either. Ultimately, as long as we can compute how judge accuracy maps to evaluation accuracy, we can infer judge accuracy from the cost ratio and the optimal number of judges.

Adjusting to the group. We imagine that after the first round of scoring and subsequent discussion, each judge scores the grants A and B again such that the new score is a mixture of the judge's original score and the panel's mean. We label the panel's mean score for grant A as μ_A and the panel's mean score for grant B as μ_B. Thus, judge i whose pre-score for grant A was S_{A_i} would score grant A after group discussion as $(z\mu_A + (1-z)S_{A_i})$, where z is a constant between 0 and 1 and represents the extent that the second score is influenced by the panel's mean. While this scheme modifies the individual scores of the judges, the sum of the judges' scores for both grants remains the same (for n judges, $\sum_{j=1}^{n}(z\mu_A + (1-z)S_{A_j}) = \sum_{j=1}^{n}(z\mu_A) + \sum_{j=1}^{n}(1-z)S_{A_j} = zn\mu_A + (1-z)n\mu_A = n\mu_A$). As such, this model does not change evaluation accuracy.

Adjusting to a random individual. In this case the reviewers score, discuss, and then score again by aligning their personal scores closer to one individual judge, a more outspoken or respected member of the group. Since the judges are all assumed to have equal accuracy, we randomly pick an individual. This time judge i whose pre-score for grant A was S_{A_i} would score grant A after group discussion as $(zS_{A_j} + (1-z)S_{A_i})$, where z is again a constant between 0 and 1 and S_{A_j} is the influential judge's score. For any one evaluation, the updated scores would result in a different sum of scores for each grant and potentially a different decision. This consultation model actually detracts from the accuracy of the evaluation because it mixes the sum of scores distribution for A and B with the less accurate distributions of a single judge.

Adjusting to a specific set of peers. Finally we consider a model with a specific pattern of judge dependency. As before the judges score, discuss, and score again. But rather than adjusting the scores according to the group's mean or a random individual we assume each judge's second score is a linear combination of other judges' scores. Furthermore, each judge is influenced by a different set of judges. In this model, judge i would score grant A after discussion as $(\sum_{j=1}^{n} z_{i,j}S_{A_j})$ while judge k would score grant A $(\sum_{j=1}^{n} z_{k,j}S_{A_j})$. Depending on how the values in the z matrix are chosen we could get very different results in evaluation

accuracy and the effects of consultation on judge accuracy. Whatever assumptions we use to produce the z matrix we can calculate post-discussion scores from pre-scores. Furthermore, since z is a matrix we can invert it to transform post-discussion scores into the initial pre-discussion scores. Thus, we can estimate the judge accuracy both before and after consultation.

Paradox example in a more complex evaluation

We expect the paradox shown in Figure 3C to appear in many different evaluation settings because if the accuracy of the judges is no better than random selection or if the accuracy is 100% then the most cost effective strategy is to use only one judge. To illustrate the paradox in a more complex system, we construct a hypothetical example with arbitrarily chosen parameters that employs the sum rule as the evaluation method, dependent scoring, and unequal judge accuracy.

In this example, we assume the reviewers are deciding between two grants: A and B. The decision will ultimately be based on the sum of scores where the grant with the highest total score will win. We assume that A is the better grant and thus can measure judge accuracy as the probability that a judge scores A higher than B. Likewise, the panel's accuracy is reflected by how often the sum of the judges' scores for A is higher than for B.

The scoring will be done in a two-stage process. First, each judge will assign scores to both grants independently, following the exact same algorithm as described in the Material and Methods section "Sum Rule vs Majority Rule Methods". Then we envision a consultation process that results in a second round of scoring. In this second round, each judge is influenced by the average scores such that each judge's scoring distribution for both grant A and grant B is adjusted based on the average score given to B (μ_B) and A (μ_A) and the judge's own personal scores from the first round. So if in the first round, judge$_i$ scored grant A with score S_{A_i} from a normal distribution N_{A_i} and grant B with score S_{B_i} from a normal distribution N_{B_i} then in the second round judge$_i$ scores grant A with a score from a normal distribution with mean $(.8S_{A_i} + .2\mu_A)$ and scores grant B with a score from a normal distribution with mean $(.8S_{B_i} + .2\mu_B)$. Thus, the judges are influenced 20% by the group and 80% by their original scores. Unlike the first example investigated under "Grant Review Examples with Dependencies" in the Supplementary material, the judges change their scoring distributions in the second round– i.e. it is not a simple linear transformation of the score before discussion.

To model the different accuracies of judges we create a hypothetical list of judge accuracies in the order they would be consulted– in this case the order is decreasing accuracy (Judge accuracies = $\{judge_1 = .80,\ judge_2 = .80,\ judge_3 = .80,\ judge_4 = .75,\ judge_5 = .75,\ judge_6 = .70,\ judge_7 = .70,\ judge_8 = .65,\ judge_9 = .65,\ judge_{10} = .60,\ judge_{11} = .60,\ judge_{12} = .60,\ judge_{13} = .55,\ judge_{14} = .55,\ judge_{15} = .55\}$). For a panel of judges, we sample the two round scoring regime $500,000$ times to calculate the probability of error (the number of times grant B gets the higher sum of scores after the two round process). We can then compute the judge accuracy curve.

In the paper, Figure 3C shows the minimal number of judges as a function of the cost ratio ($C_{\text{error}}/C_{\text{judge}}$) and judge accuracy. Since, in this example we have a mixed panel there is not one judge accuracy. However, we imagine that the judges can be "trained" such that the accuracy of the panel, every judge, increases. Because we calculate accuracy using the sum rule as the overlap between scoring distributions for grant A and grant B, we imagine that the training regime further separates the distributions for every judge. In the paper, a judge's mean score for grant A was set to be a factor "c" times the mean score for grant B. To effect training, we simply increased "c" by a constant which further separated the scoring distributions for A and B. We then can compute the judge accuracy curve for panels of different amounts of training. We use this information to find the optimal number of judges to reduce the total cost (see Figure S1).

Code for Figures

Code for Figure 1b (in Matlab).

```
InitialPanelNum=3;
InitialPanelAcc=.8;
ExtraJudges=[0:2:24];
J60curve=zeros(size(ExtraJudges));
J70curve=zeros(size(ExtraJudges));
J80curve=zeros(size(ExtraJudges));
for k2=0:1:InitialPanelNum
    Prob=nchoosek(InitialPanelNum,k2)*InitialPanelAcc^k2*(1-InitialPanelAcc)^(InitialPanelNum-k2);
    for k1=1:length(ExtraJudges)
        MajNum=ceil((InitialPanelNum+ExtraJudges(k1))/2);
        for k3=max([(MajNum-k2) 0]):1:ExtraJudges(k1)
            J60curve(k1)=J60curve(k1)+nchoosek(ExtraJudges(k1),k3)*.6^k3*(1-.6)^(ExtraJudges(k1)-k3)*Prob;
            J70curve(k1)=J70curve(k1)+nchoosek(ExtraJudges(k1),k3)*.7^k3*(1-.7)^(ExtraJudges(k1)-k3)*Prob;
            J80curve(k1)=J80curve(k1)+nchoosek(ExtraJudges(k1),k3)*.8^k3*(1-.8)^(ExtraJudges(k1)-k3)*Prob;
        end
    end
end

% The three curves plotted in Fig. 1b are simply J60curve, J70curve, and J80curve versus ExtraJudges
% Below are the commands to plot the figure

plot(ExtraJudges,J60curve,'r',ExtraJudges,J70curve,'b',ExtraJudges,J80curve,'g','LineWidth',5)
hold on;
plot(ExtraJudges,J60curve,'ko',ExtraJudges,J70curve,'ko',ExtraJudges,J80curve,'ko','LineWidth',5)
axis([-1.5 25.5 .80 1.01]);
set(gca,'xTick',[0:2:24],'yTick', [.8:.05:1],'TickLength',[.02,.02],'LineWidth',3)
axis square
```

Code for Figure 2c (in Matlab).

```
% Majority Rule Curve

Acc70=.70;
NumJudges=[1:2:25];
MajAcc70=zeros(size(NumJudges));
for k1=1:length(NumJudges);
    MajNum=ceil(NumJudges(k1)/2);
    for k2=MajNum:1:NumJudges(k1)
```

```
          MajAcc70(k1)=MajAcc70(k1)+nchoosek(NumJudges(k1),k2)*Acc70^k2*(1-Acc70)^(NumJudges(k1)-k2);
     end
end

% Sum Rule Curve
NumJudges=[1:2:25];
Std=.7;
Mean=7;
C=1.16;
Num=100000;
SumAcc70=zeros(size(NumJudges));
for k1=1:length(NumJudges);
     MnB=Mean+Std*randn(Num,NumJudges(k1));
     MnA=C*MnB;
     ScoresA=MnA+.2*MnA.*randn(Num,NumJudges(k1));
     ScoresB=MnB+.2*MnB.*randn(Num,NumJudges(k1));
     SumAcc70(k1)=(sum(sum(ScoresA,2)>sum(ScoresB,2)))/Num;
end;

% The curves plotted in the figure are MajAcc70 and SumAcc70,
% Below are the commands to plot the figure

plot(NumJudges,MajAcc70,'b',NumJudges,SumAcc70,'r','LineWidth',5)
hold on;
plot(NumJudges,MajAcc70,'ko',NumJudges,SumAcc70,'ko','LineWidth',5)
axis([0 26 .65 1.025]);
set(gca,'xTick',[1:2:25],'yTick', [.7:.05:1.0],'TickLength',[.02,.02],'LineWidth',3)
axis square
```

Code for Figure 2e (in Matlab).

```
% Majority Rule Curves
Acc65=.65;
Acc70=.70;
NumJudges=[1:2:25];
MajAcc65=zeros(size(NumJudges));
MajAcc70=zeros(size(NumJudges));
for k1=1:length(NumJudges);
     MajNum=ceil(NumJudges(k1)/2);
     for k2=MajNum:1:NumJudges(k1)
          MajAcc65(k1)=MajAcc65(k1)+nchoosek(NumJudges(k1),k2)*Acc65^k2*(1-Acc65)^(NumJudges(k1)-k2);
          MajAcc70(k1)=MajAcc70(k1)+nchoosek(NumJudges(k1),k2)*Acc70^k2*(1-Acc70)^(NumJudges(k1)-k2);
     end
end

% Sum Rule curve
NumJudges=[1:2:25];
Sigma=(sqrt(log(1400^2/700^2+1)));
Mu=log(700)-Sigma^2/2;
C=1.16;
SumAcc70=zeros(size(NumJudges));
Num=100000;
for k1=1:length(NumJudges);
     MnB=exp(Mu+Sigma*randn(Num,NumJudges(k1)));
     MnA=C*MnB;
     ScoresA=MnA+.2*MnA.*randn(Num,NumJudges(k1));
     ScoresB=MnB+.2*MnB.*randn(Num,NumJudges(k1));
     SumAcc70(k1)=(sum(sum(ScoresA,2)>sum(ScoresB,2)))/Num;
end;

% The curves plotted in the figure are MajAcc70, SumAcc70, and MajAcc65,
% Below are the commands to plot the figure

plot(NumJudges,MajAcc70,'b',NumJudges,SumAcc70,'r',NumJudges,MajAcc65,'g','LineWidth',5);
hold on;
plot(NumJudges,MajAcc70,'ko',NumJudges,SumAcc70,'ko',NumJudges,MajAcc65,'ko','LineWidth',5);
axis([0 26 .65 1.025]);
```

```
set(gca,'xTick',[1:2:25],'yTick', [.7:.05:1.0],'TickLength',[.02,.02],'LineWidth',3)
axis square
```

Code for Figure 3a (in Matlab).

```
% Majority Rule Curves
Acc65=.65;
Acc70=.70;
NumJudges=[1:2:25];
MajAcc65=zeros(size(NumJudges));
MajAcc70=zeros(size(NumJudges));
for k1=1:length(NumJudges);
     MajNum=ceil(NumJudges(k1)/2);
     for k2=MajNum:1:NumJudges(k1)
          MajAcc65(k1)=MajAcc65(k1)+nchoosek(NumJudges(k1),k2)*Acc65^k2*(1-Acc65)^(NumJudges(k1)-k2);
          MajAcc70(k1)=MajAcc70(k1)+nchoosek(NumJudges(k1),k2)*Acc70^k2*(1-Acc70)^(NumJudges(k1)-k2);
     end
end

% Sum Rule Curve
NumJudges=[1:2:25];
Sigma=(sqrt(log(1400^2/700^2+1)));
Mu=log(700)-Sigma^2/2;
C=1.16;
SumAcc70=zeros(size(NumJudges));
Num=1000000;
for k1=1:length(NumJudges);
     MnB=exp(Mu+Sigma*randn(Num,NumJudges(k1)));
     MnA=C*MnB;
     ScoresA=MnA+.2*MnA.*randn(Num,NumJudges(k1));
     ScoresB=MnB+.2*MnB.*randn(Num,NumJudges(k1));
     SumAcc70(k1)=(sum(sum(ScoresA,2)>sum(ScoresB,2)))/Num;
end;

% Inclusion of Cost
Cjudge=1;
Cerror=55*Cjudge;
CostSum70=Cjudge*NumJudges+Cerror*(1-SumAcc70);
CostMaj70=Cjudge*NumJudges+Cerror*(1-MajAcc70);
CostMaj65=Cjudge*NumJudges+Cerror*(1-MajAcc65);

% Below are the commands to plot the figure

plot(NumJudges,CostMaj70,'b',NumJudges,CostSum70,'r',NumJudges,CostMaj65,'g','LineWidth',5)
hold on;
plot(NumJudges,CostMaj70,'ko',NumJudges,CostSum70,'ko',NumJudges,CostMaj65,'ko','LineWidth',5)
[u,v]=min(CostMaj70);
plot(NumJudges(v),CostMaj70(v),'ko','LineWidth',8);
[u,v]=min(CostSum70);
plot(NumJudges(v),CostSum70(v),'ko','LineWidth',8);
[u,v]=min(CostMaj65);
plot(NumJudges(v),CostMaj65(v),'ko','LineWidth',8);
axis([0 26 12 30]);
set(gca,'xTick',[1:2:25],'yTick', [14:4:30],'TickLength',[.02,.02],'LineWidth',3)
axis square
```

Code for Figure 3b (in Matlab).

```
Acc1=.65;
Acc2=.7;
NumJudges=[1:2:25];
Prob1=zeros(size(NumJudges));
Prob2=zeros(size(NumJudges));
for k1=1:length(NumJudges)
     MajNum=ceil(NumJudges(k1)/2);
     for k2=MajNum:1:NumJudges(k1)
          Prob1(k1)=Prob1(k1)+nchoosek(NumJudges(k1),k2)*Acc1^k2*(1-Acc1)^(NumJudges(k1)-k2);
          Prob2(k1)=Prob2(k1)+nchoosek(NumJudges(k1),k2)*Acc2^k2*(1-Acc2)^(NumJudges(k1)-k2);
```

```
     end
end

CostErrors=[1:.01:150];
CostJudge=1;
OptJudge1=zeros(size(CostErrors));
OptJudge2=zeros(size(CostErrors));
for k1=1:length(CostErrors);
     Cost1=CostErrors(k1)*(1-Prob1)+CostJudge*NumJudges;
     Cost2=CostErrors(k1)*(1-Prob2)+CostJudge*NumJudges;
     [u,v]=min(Cost1);
     OptJudge1(k1)=NumJudges(v);
     [u,v]=min(Cost2);
     OptJudge2(k1)=NumJudges(v);
end

% The green and blue plots are OptJudge1 and OptJudge2, respectively.
% Below are the commands to plot the figure

inds=find(abs(OptJudge1-OptJudge2)<.1);
plot(CostErrors,OptJudge1,'go-',CostErrors,OptJudge2,'bo-',CostErrors(inds),OptJudge2(inds),'ko','LineWidth',5);
set(gca,'yTick',[1:2:17],'xTick',[0:25:150],'TickLength',[.02,.02],'LineWidth',3)
axis([0 155 0 18])
axis square
```

Code for Figure 3c (in Matlab).

```
NumJudges=[1:2:25];
Accs=[.51:.01:.99]';
CostRatios=[.1:.1:150];
OptJudge=zeros(length(Accs),length(CostRatios));
for k1=1:length(Accs);
     PanelAcc=zeros(size(NumJudges));
     for k2=1:length(NumJudges);
          MajNum=ceil(NumJudges(k2)/2);
          for k3=MajNum:1:NumJudges(k2)
               PanelAcc(k2)=PanelAcc(k2)+nchoosek(NumJudges(k2),k3)*Accs(k1)^k3*(1-Accs(k1))^(NumJudges(k2)-k3);
          end
     end
     for k2=1:length(CostRatios)
          CostError=CostRatios(k2);
          CostJudge=1;
          Cost=CostError*(1-PanelAcc)+CostJudge*NumJudges;
          [u,v]=min(Cost);
          OptJudge(k1,k2)=NumJudges(v);
     end
end

% Below are the commands to plot the figure

contour(CostRatios,Accs,OptJudge,'LineWidth',3);
axis([0 151 .5 1]);
axis square
set(gca,'xTick',[0:25:150])
```
