# Supplementary figures and images for: The Calculus of Committee Composition
Source: PLoS One. 2010 Sep 17;5(9):e12642. doi: 10.1371/journal.pone.0012642 (PMC2943248; doi:10.1371/journal.pone.0012642)

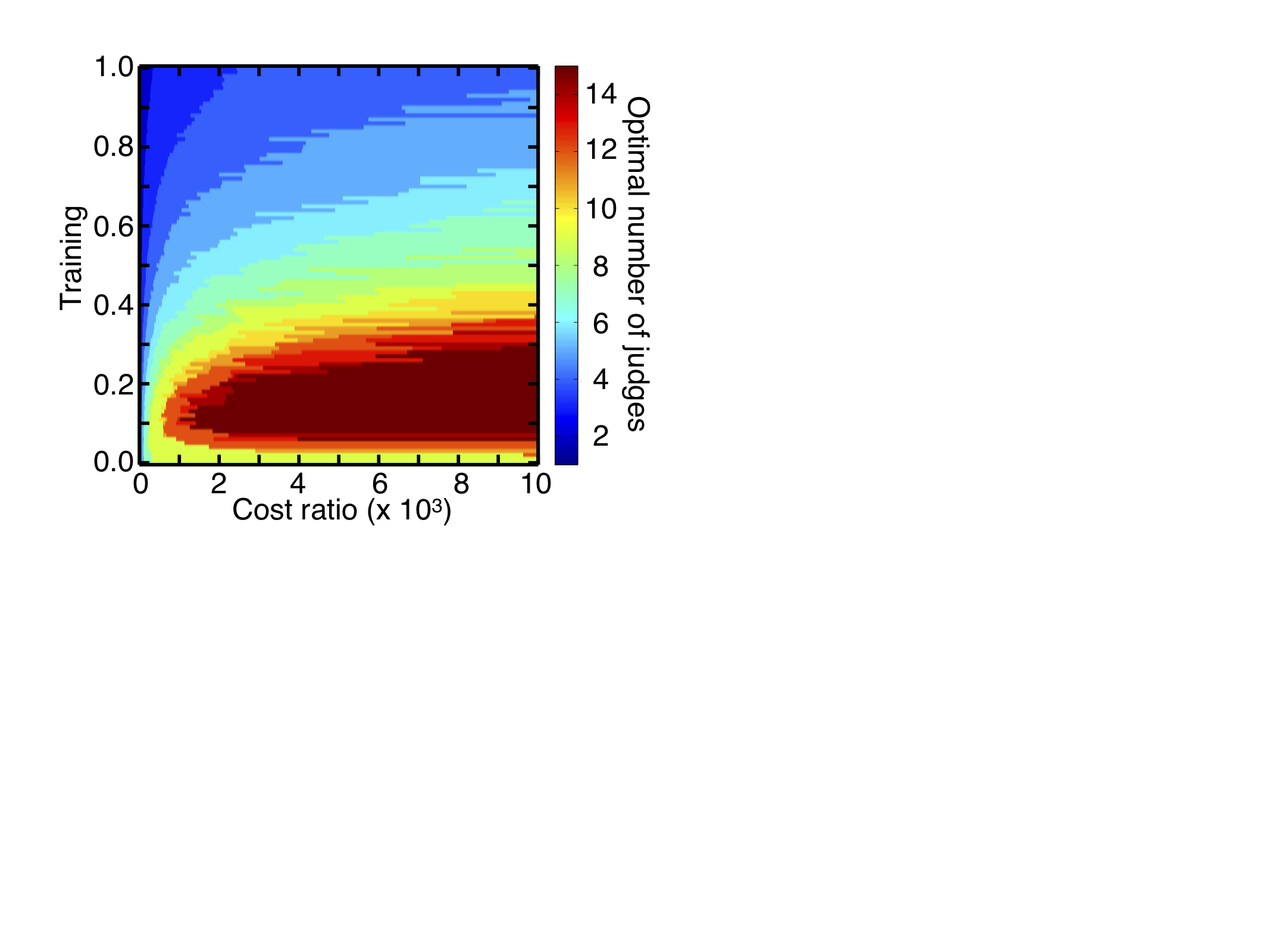

Supplement: Figure S1 — The vertical axis is the constant added to “c” for every judge, thus further separating the scoring distributions for the grants and improving each judge's accuracy. The horizontal axis is the cost ratio, (cost per error)/ (cost per judge). The colored bar shows the optimal number of judges. As in Figure 3C from the paper, there is a single peaked surface, and thus the paradox is present. (0.15 MB TIF) [file pone.0012642.s002.tif]
